# Maxillary Expansion

**DOI:** 10.5005/jp-journals-10005-1069

**Published:** 2010-09-15

**Authors:** Anirudh Agarwal, Rinku Mathur

**Affiliations:** 1 Professor and Head, Department of Orthodontics, Rajasthan Dental College and Hospital, Jaipur, Rajasthan, India; 2 Assistant Professor, Department of Pedodontics and Preventive Dentistry, Government Dental College and Hospital, Jaipur Rajasthan, India

**Keywords:** Maxillary expansion, Rapid maxillary expansion, Slow maxillary expansion, Surgically assisted maxillary expansion.

## Abstract

Maxillary transverse discrepancy usually requires expansion of the palate by a combination of orthopedic and orthodontic tooth movements. Three expansion treatment modalities are used today: rapid maxillary expansion, slow maxillary expansion and surgically assisted maxillary expansion.This article aims to review the maxillary expansion by all the three modalities and a brief on commonly used appliances.

## INTRODUCTION

Maxillary expansion treatments have been used for more than a century to correct maxillary transverse deficiency. The earliest common cited report is that of E.C. Angell published in *Dental Cosmos* in 1860.^1^ The work was discredited at the time, but the technique is now generally accepted as a relatively simple and predictable orthodontic therapy. Correction of the transverse discrepancy usually requires expansion of the palate by a combination of orthopedic and orthodontic tooth movements. Three expansion treatment modalities are used today: rapid maxillary expansion (RME), slow maxillary expansion (SME) and surgically assisted maxillary expansion. Since each treatment modality has advantages and disadvantages, controversy regarding the use of each exists. Practitioners select treatment appliances based on their personal experiences and on the patient’s age and malocclusion.^2,3^ Normal palatal growth is nearly complete by age 6,^9^ and increasing interdigitation of the suture makes separation difficult to achieve after puberty.^10-15^ During treatment, transverse forces tip the buccal segments laterally^4^ and with proper appliance design, 3rd-order moments will induce bodily translation.^5-8^ If the force is strong enough, separation occurs at the maxillary suture. The clinical conditions indicating maxillary expansion include crossbites, distal molar movement, functional appliance treatment, surgical cases for instance arch coordination or bone grafts, to aid maxillary protraction and mild crowding. This article aims to review the maxillary expansion and commonly used appliances.

## RAPID MAXILLARY EXPANSION (RME)

Rapid maxillary expansion was first described by Emerson Angell in 1860^1^ and later repopularized by Haas. The main object of RME is to correct maxillary arch narrowness but its effects are not limited to the maxilla as it is associated with 10 bones in the face and head.^16^ Advocates of rapid maxillary expansion believe that it results in minimum dental movement (tipping) and maximum skeletal movement.^3^ When heavy and rapid forces are applied to the posterior teeth, there is not enough time for tooth movement to occur and the forces are transferred to the sutures. When the force delivered by the appliance exceeds the limit needed for orthodontic tooth movement and sutural resistance, the sutures open up while the teeth move only minimally relative to their supporting bone. The appliance compresses the periodontal ligament, bends the alveolar process, tips the anchor teeth, and gradually opens the midpalatal suture and all the other maxillary sutures.

### Effect of RME on Maxillary and Mandibular Complex

*Maxillary skeletal effect:* When viewed occlusally, Inoue found that the opening of the midpalatine suture was nonparallel and triangular with maximum opening at incisor region and gradually diminishing towards the posterior part of palate.

Viewed frontally, the maxillary suture separates supero-inferiorly in a nonparallel manner.^17^ It is pyramidal in shape with the base of pyramid located at the oral side of the bone.

*Maxillary halves:* Haas^18^ and Wertz^19^ found the maxilla to be frequently displaced downward and forward.

*Palatal vault:* Haas^18^ reported that the palatine process of maxilla was lowered as a result of outward tilting of maxillary halves.

*Alveolar process:* Because bone is resilient, lateral bending of the alveolar processes occurs early during RME, which rebounds back after a few days.^20^

*Maxillary anterior teeth:* From the patient’s point of view, one of the most spectacular changes accompanying RME is the opening of a diastema between the maxillary central incisors. It is estimated that during active suture opening, the incisors separate approximately half the distance the expansion screw has been opened,^18^ but the amount of separation between the central incisors should not be used as an indication of the amount of suture separation.^19^ This distema is self-corrective due to elastic recoil of the transseptal fibers.

*Maxillary posterior teeth:* There is buccal tipping and extrusion of the maxillary molars.

The posterior maxilla expands less readily because of the resistance produced by the zygomatic buttress and pterygoid plates.

*Effect of RME on mandible:* There is a concomitant tendency for the mandible to swing downward and backward. ^17^

*RME and nasal airflow:* Anatomically, there is an increase in the width of the nasal cavity immediately following expansion thereby improves in breathing. The nasal cavity width gain averages of 1.9 mm, but can be as wide as 8 to 10 mm.^21^

It is important for the clinician to remember that the main resistance to midpalatal suture opening is probably not the suture itself, but in the surrounding structures particularly the sphenoid and zygomatic bones. ^17^

## INDICATIONS/CONTRAINDICATIONS OF RME

Rapid maxillary expansion is indicated in cases with a transverse discrepancy equal to or greater than 4 mm, and where the maxillary molars are already buccally inclined to compensate for the transverse skeletal discrepancy. Rapid palatal expansion has been used to facilitate maxillary protraction in class III treatment by disrupting the system of sutures, which connect the maxilla to the cranial base, cleft lip and palate patients with collapsed maxillae are also RME candidates. Finally, some clinicians use the procedure to gain arch length in patients, who have moderate maxillary crowding. It is contraindicated in patients, who have passed the growth spurt, have recession on the buccal aspect of the molars, anterior open bite, steep mandibular plane, convex profiles and who show poor compliance.

It appears that approximately 1 millimeter per week is the maximum rate at which the tissue of the midpalatal suture can adapt, so that tearing and hemorrhaging are minimized compared with rapid expansion protocols. The amount of orthopedic vs. orthodontic change depends greatly on the patient’s age. Normal palatal growth is nearly complete by age 6,^9^ and increasing interdigitation of the suture makes separation difficult to achieve after puberty.^10-15^ RPE appliances require frequent activations and generate heavy forces―as much as 2-5 kg per quarter-turn with accumulated loads of more than 9 kg.^22^ The disadvantages of using rapid palatal expanders include discomfort due to heavy forces used, traumatic separation of the midpalatal suture, inability to correct rotated molars, requirement of patient or parent cooperation in activation of the appliance, bite opening, relapse, microtrauma of the temporomandibular joint and midpalatal suture, root resorption, tissue impingement, pain and laborintensive procedure in fabrication of the appliance.

## CLINICAL MANAGEMENT OF RME

The patient/parent should be informed in advance about the upper midline diastema during the expansion phase. This is likely to close spontaneously during the retention period. Patients should be instructed to turn the expansion screw one-quarter turn twice a day (am and pm). This may be associated with minor discomfort. Force levels tend to accumulate following multiple turns and can be as high as 10 kg following many turns. Patients should be reviewed weekly and some clinicians recommend that an upper occlusal radiograph be taken one week into treatment to ensure that the midpalatal suture has separated. If there is no evidence of this, it is important to stop appliance activation as there is a risk of alveolar fracture and/or periodontal damage. Active treatment is usually required for a period of 2-3 weeks, after which a retention period of three months is recommended to allow for bony infilling of the separated suture.^23^

## APPLIANCES FOR RME

These are banded and bonded appliances. The banded appliance are attached to teeth with bands on the maxillary first molar and first premolars. The banded appliances are hygienic as there is no palatal coverage. The banded RME are of two types:

1. Tooth and tissue borne (Fig. 1A)
2. Tooth borne (Fig.1B).

## TOOTH BORNE RME

They consist of only bands and wires without any acrylic covering.

1. *HYRAX expander:* It is a tooth borne appliance, which was introduced by William Biederman in 1968. This type of appliance makes use of a special screw called HYRAX (Hygenic Rapid Expander). The Hyrax Expander (Fig. 2) is essentially a nonspring loaded jackscrew with an all wire frame.^24^ The screws have heavy gauge wire extensions that are adapted to follow the palatal contours and soldered to bands on premolar and molar. The main advantage of this expander is that it does not irritate the palatal mucosa and is easy to keep clean. It is capable of providing sutural separation of 11 mm within a very short period of wear and a maximum of 13 mm can also be achieved. Each activation of the screw produces approximately 0.2 mm of lateral expansion and it is activated from front to back.
2. *Issacson expander:* It is a tooth borne appliance without any palatal covering. This expander makes use of a spring loaded screw called Minne expander (developed by university of Minnesota, dental school), which is soldered directly to the bands on first premolar and molars.^24^ The Minne expander is a heavily calibrated coil spring expanded by turning a nut to compress the coil. Two metal flanges perpendicular to the coil are soldered to the bands on abutment teeth. The Minne expander may continue to exert expansion forces after completion of the expansion phase unless they are partly deactivated.

## TOOTH AND TISSUE BORNE RME

They consist of an expansion screw with acrylic abutting on alveolar ridges. Haas, in 1970, gave the following advantages of tooth and tissue RME:

1. Produces more parallel expansion
2. Less relapse
3. Greater nasal cavity and apical base gain
4. More favorable relationship of the denture bases in width and frequently in the anteroposterior plane as well
5. Creates more mobility of the maxilla instead of teeth.

**Fig. 1A:**
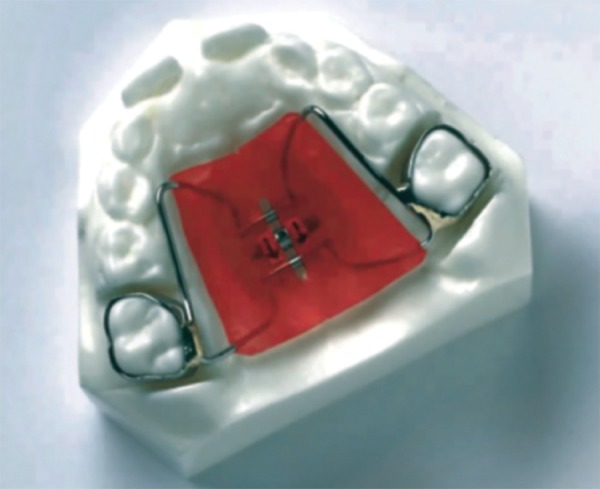
Tooth and tissue borne

**Fig. 1B:**
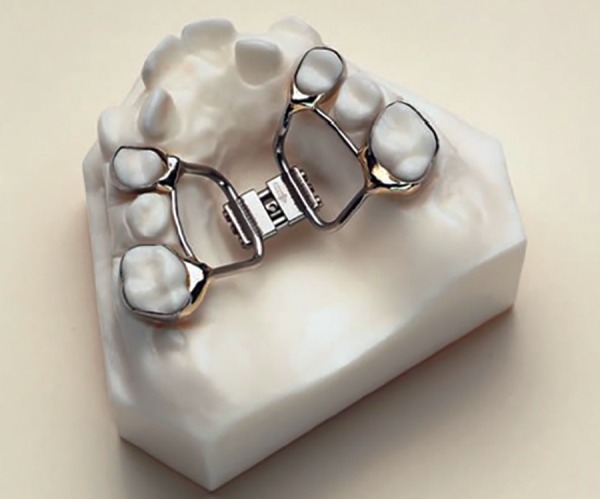
Tooth borne

**Fig. 2:**
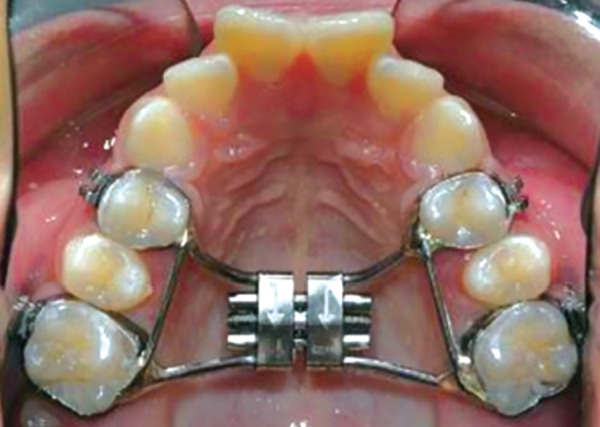
HYRAX expander

Disadvantage of Tooth and Tissue Borne RME

These tooth and tissue borne RME tend to have higher soft tissue irritation.

Types of Tooth and Tissue Borne RME

**Fig. 3:**
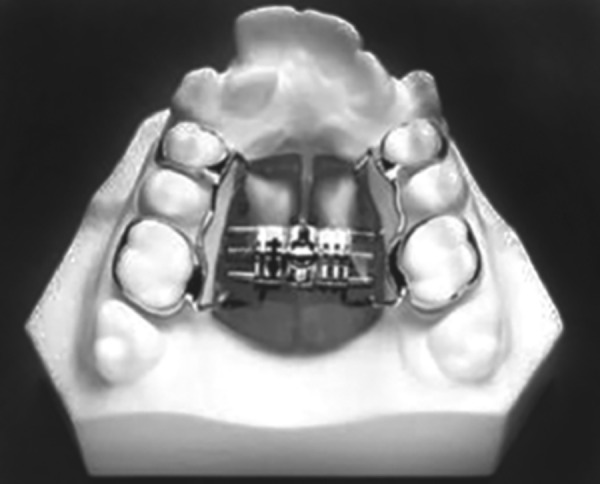
Haas expander

1. *Haas:* The basis for the rapid expansion procedure is to produce immediate midpalatal suture separation by disruption of the sutural connective tissue (Fig. 3). The rapid palatal expander as described by Haas is a rigid appliance designed for maximum dental anchorage that uses a jackscrew to produce expansion in 10 to 14 days.^14^ He believed that this will maximize the orthopedic effects and forces produced by this appliance have been reported in the range of 3 to 10 pounds.
2. *Derichsweiler:* The first premolar and molars are banded. Wire tags are soldered to these bands and then inserted to the split palatal acrylic, which contains the screw.

## BONDED RAPID PALATAL EXPANDER

**Fig. 4:**
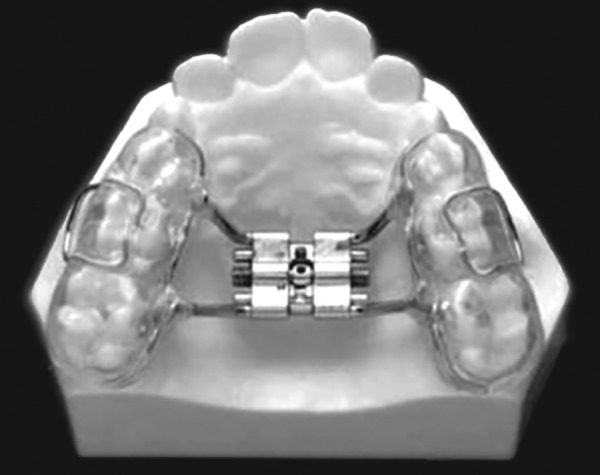
Bonded palatal expander

The Bonded RPE were first described by Cohen and Silverman in 1973 (Fig. 4). It is similar to the banded version with the exception of the method of attachment to the teeth. This appliance is constructed with an acrylic cap over the posterior segments, which is then bonded directly to the teeth.^25^ The bonded appliance has become increasingly popular because of its advantages:

1. It can be easily cemented during the mixed dentition stage, when retention from other appliances can be poor.
2. Number of appointments are reduced.
3. There is reduced posterior teeth tipping and extrusion. The buccal capping limits molar extrusion during treatment and, therefore improves the vertical control, which is particularly useful in class II conditions, as molar extrusion would cause autorotation of the mandible backward and downward resulting in increase in facial convexity and the vertical dimension of the lower face.^25^
4. It provides Bite block effect to facilitate the correction of anterior crossbite (McNamara).^26^

## IPC RAPID PALATAL EXPANDER

**Fig. 5:**
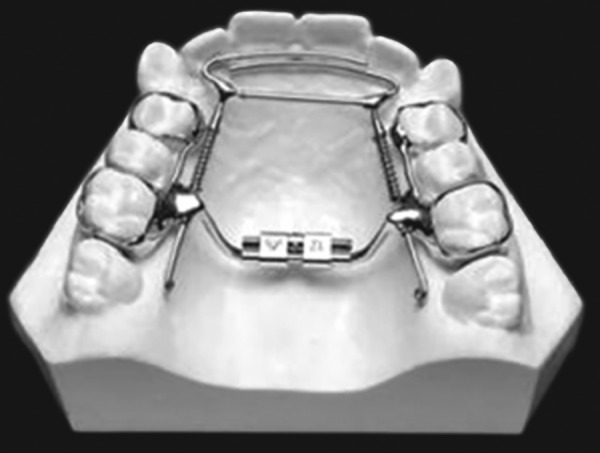
IPC palatal expander

IPC is designed for orthopedic expansion along with labial alignment of incisors (Fig. 5). As expansion occurs, the IPC controls the NiTi open coil spring force applied to the lingual surface of the anterior teeth. Wire around the distal end of the lateral incisors limits the midline diastema that often occurs during RPE treatment.

## SLOW MAXILLARY EXPANSION (SME)

SME procedures produce less tissue resistance around the circummaxillary structures and, therefore improve bone formation in the intermaxillary suture, which theoretically should eliminate or reduce the limitations of RME.

Slow expansion has been found to promote greater post-expansion stability,^5,8,15,22^ if given an adequate retention period. It delivers a constant physiologic force until the required expansion is obtained. The appliance is light and comfortable enough to be kept in place for sufficient retention of the expansion. Prefabrication eliminates extra appointments for impressions and the time and expense of laboratory fabrication.

For SME, 10 to 20 newtons of force should be applied to the maxillary region only 450 to 900 gm of force is generated, which may be insufficient to separate a progressively maturing suture.^8,9,14,15,18,23,27^ Maxillary arch-width increases ranged from 3.8 to 8.7 mm with slow expansion of as much as 1 mm per week using 900 gm of force.^15^

## APPLIANCES IN SME

### Coffin Appliance

Given by Walter Coffin-1875. It is a removable appliance capable of slow dento alveolar expansion. The appliance consists of an omega-shaped wire of 1.25 mm thickness, placed in the midpalatal region. The free ends of the omega wire are embedded in acrylic covering the slopes of the palate. The spring is activated by pulling two asides apart manually.

### Magnets

Repulsive magnetic forces for maxillary expansion were first described by Vardemon et al 19 87.^28^ Banded magnets produced more pronounced skeletal; versus overall expansion effects. The continuous force of 250-500 gm could generate dental and skeletal movements, the degree depending on patients status (age, growth, etc). Disadvantage of magnets is that they tend to be oxidized in the oral environment due to the potential formation of corrosive products but this can be overcome by coating magnets. The advantage of these magnets is that they impart measured continuous force over a long period of time, hence the risk of external root resorption is decreased. These magnets are quite bulky as they must be adequately stabilized and contain stout guide rods to prevent the magnets becoming out of line and causing unwanted rotational movements.

### W-Arch

**Fig. 6:**
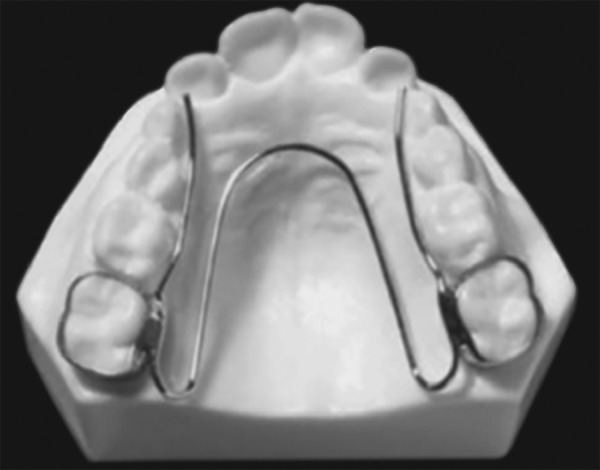
W-arch

The “W”; expansion appliance was originally used by Ricketts and his colleagues^29^ to treat cleft palate patients (Fig. 6). The W-arch is a fixed appliance constructed of 36 mil steel wire soldered to molar bands. To avoid soft tissue irritation, the lingual arch should be constructed so that it rests 1-1.5 mm off the palatal soft tissue. It is activated simply by opening the apices of W-arch and is easily adjusted to provide more anterior than posterior expansion, or *vice versa* if this is desired. The appliance delivers proper force levels when opened 3-4 mm wider than the passive width and should be adjusted to this dimension before being inserted. Expansion should continue at the rate of 2 mm per month until the cross bite is slightly overcorrected.

### Quadhelix

**Fig. 7:**
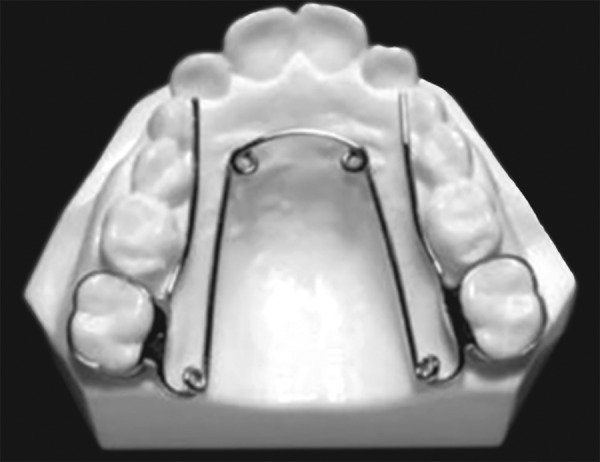
Quadhelix

The quadhelix appliance is a modification of Coffin’s W-spring and was described by Ricketts (Fig. 7). The incorporation of four helices into the W-spring helped to increase the flexibility and range of activation. The length of the palatal arms of the appliance can be altered depending upon which teeth arch in crossbite. A new generation of prefabricated appliances, constructed from nickel titanium, have been introduced more recently. The advantages of using nickel titanium over stainless steel include its more favorable force delivery characteristics as it has superelastic properties. This may help to produce more physiological tooth movement with more rapid correction of crossbites.

Mode of Action

The quadhelix appliance works by a combination of buccal tipping and skeletal expansion in a ratio of 6:1 in prepubertal children.

Clinical Management

The desirable force level of 400 gm can be delivered by activating the appliance by 8 mm, which equates to approximately one molar width. Patients should be reviewed on a six-weekly basis.^27^ Sometimes, the appliance can leave an imprint on the tongue, however this will rapidly disappear following treatment. Expansion should be continued until the palatal cusps of the upper molars meet edge-to-edge with the buccal cusps of the mandibular molars. A degree of overcorrection is desirable as relapse is inevitable. A three-month retention period, with the quadhelix in place, is recommended once expansion has been achieved. If fixed appliances are being used, the quadhelix can be removed once stainless steel wires are in place.

*Advantages:* Good retention, a large range of action, orthopedic effect, differential expansion, habit breaker, fixed appliances can be incorporated, molar rotation/torque, non-compliance and cost-effective.

*Disadvantages:* Molar tipping, bite opening, limited skeletal change.

### Spring Jet

**Fig. 8:**
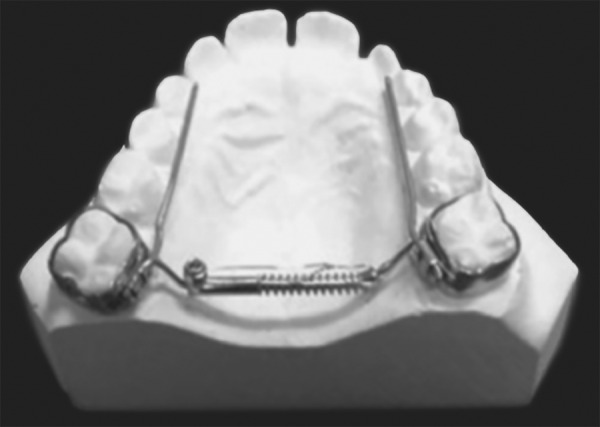
Spring jet

The active components of the spring jet are soldered or attached to the molar bands (Fig. 8). The telescopic unit is placed upto 5 mm from center of molar tubes so that the forces pass close to the center of resistance of maxillary teeth, but it should be 1.5 mm away from palatal tissue. Force applied in mixed dentition is 240 gm and 400 gm in the permanent dentition. Activation is done by moving the lock screw horizontally along the telescopic tube. A ball stop on the transpalatal wire allows the spring to be compressed.

### NiTi Expander

**Fig. 9:**
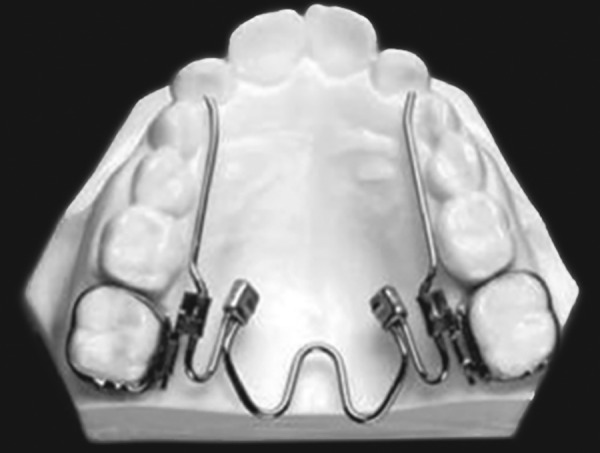
NiTi expander

The Nickel Titanium Palatal Expanders were introduced by Wendell V^30^ (Fig. 9). It generates optimal, constant expansion forces. The central component is made of a thermally activated NiTi alloy and rest of component is made of stainless steel. The expander may be used simultaneously with conventional fixed appliances, requiring only an additional lingual sheath on the molar bands.

The action of the appliance is a consequence of nicket titanium’s shape memory and transition temperature effects.^31^ The nickel titanium component has a transition temperature of 94° F. At room temperature, the expander is too stiff to bend for insertion. Chilling the expander softens the central component allowing easy manipulation. Once placed, stiffens and begins to return to its original shape. A 3 mm increment of expansion exerts only about 350 gm of force^31^ and the nickel titanium alloy provides relatively uniform force levels as the expander deactivates.

### Surgical Techniques

The effect of dental arch on the maxillary base diminishes as age advances so, surgically assisted expansion techniques can be considered. Indications of surgical expansion are:

1. To widen the arch
2. To correct posterior crossbite when large amount (>7 mm) of expansion is required to avoid the potential increased risk of segmental osteotomies
3. To widen the arch following maxillary collapse associated with a cleft palate, in cases with extremely thin and delicate gingival tissue, or presence of significant buccal gingival recession in the canine-bicuspid region of the maxilla; and in condition, where significant nasal stenosis is found.

The techniques available are:

- Surgically assisted rapid palatal expansion (SARPE)^32^
- Segmental maxillary surgery.

Surgically assisted rapid palatal expansion (SARPE) has gradually gained popularity as a treatment option to correct MTD (Maxillary Transverse Deficiency). It allows clinicians to achieve effective maxillary expansion in a skeletally mature patient.

Segmental Maxillary Surgery–transverse expansion can be produced during a Le Fort 1 osteotomy by creating an additional surgical cut along the midpalatal suture. The maxillary halves are then separated and retained in the new position. The relative inelasticity of the palatal muco-periosteum limits the degree of expansion that may be achieved.

Before surgery, orthodontic treatment involves moving the roots of the maxillary central incisors apart to improve surgical access to the osteotomy site. This is the technique of choice in patients, who require expansion and have coexisting sagittal and/or vertical maxillary discrepancies.

## CONCLUSION

Expansion of the maxilla and the maxillary dentition may be accomplished in numerous ways. The type of skeletal and dental pattern greatly influences the type of expansion chosen and the type of expansion selected can greatly facilitate the overall treatment objectives.
